# Cu/Cu_2_O nanocomposite films as a p-type modified layer for efficient perovskite solar cells

**DOI:** 10.1038/s41598-018-25975-8

**Published:** 2018-05-16

**Authors:** You-Jyun Chen, Ming-Hsien Li, Jung-Chun-Andrew Huang, Peter Chen

**Affiliations:** 1 Department of Photonics, National Cheng Kung University, Tainan, 701 Taiwan; 2 Department of Physics, National Cheng Kung University, Tainan, 701 Taiwan; 3 Center for Micro/Nano Science and Technology (CMNST), National Cheng Kung University, Tainan, 701 Taiwan; 4 Quantum Topology Center (QTC), National Cheng Kung University, Tainan, 701 Taiwan; 5 Hierarchical Green-Energy Materials Research Center, National Cheng Kung University, Tainan, 701 Taiwan

## Abstract

Cu/Cu_2_O films grown by ion beam sputtering were used as p-type modified layers to improve the efficiency and stability of perovskite solar cells (PSCs) with an n-i-p heterojunction structure. The ratio of Cu to Cu_2_O in the films can be tuned by the oxygen flow ratio (O_2_/(O_2_ + Ar)) during the sputtering of copper. Auger electron spectroscopy was performed to determine the elemental composition and chemical state of Cu in the films. Ultraviolet photoelectron spectroscopy and photoluminescence spectroscopy revealed that the valence band maximum of the p-type Cu/Cu_2_O matches well with the perovskite. The Cu/Cu_2_O film not only acts as a p-type modified layer but also plays the role of an electron blocking buffer layer. By introducing the p-type Cu/Cu_2_O films between the low-mobility hole transport material, spiro-OMeTAD, and the Ag electrode in the PSCs, the device durability and power conversion efficiency (PCE) were effectively improved as compared to the reference devices without the Cu/Cu_2_O interlayer. The enhanced PCE is mainly attributed to the high hole mobility of the p-type Cu/Cu_2_O film. Additionally, the Cu/Cu_2_O film serves as a protective layer against the penetration of humidity and Ag into the perovskite active layer.

## Introduction

Since the pioneering work of Prof. Miyasaka^1^, hybrid organic-inorganic halide perovskite materials have attracted tremendous research interest owing to their inherent photo-electrical properties, making them suitable light absorbers for photovoltaic devices. To date, the highest power conversion efficiency (PCE) of 22.7% has been demonstrated for perovskite solar cells (PSCs)^2^. The selection of hole transport materials (HTMs) is crucial for effective hole transport from the perovskite structure and the resultant photovoltaic performance^3–8^. High efficiency PSCs usually use organic HTMs such as 2,2′,7,7′-tetrakis-(N,N-di-p-methoxyphenylamine)-9,9′-bifluorene (spiro-OMeTAD), Poly(3,4-ethylenedioxythiophene)-poly(styrenesulfonate) (PEDOT:PSS), Poly(triarylamine) (PTAA), and Poly(3-hexylthiophene-2,5-diyl) (P3HT). However, their low environmental stability hampers the application of such PSCs. In contrast with organic HTMs, inorganic HTMs are of considerable interest for application in PSCs owing to their long-term stability and high mobility^6,7^. The use of inorganic HTMs on perovskites can also reduce or even prevent oxygen and water penetration, reducing the degradation rate of the as-formed perovskite^9–11^. However, the deposition of inorganic HTMs on perovskite must be performed below 100 °C, as perovskite is unstable at high temperatures^12–18^. Therefore, the appropriate preparation of HTMs is a key issue in the further development of perovskite solar cells.

Over the past few decades, the electrical, optical, and magnetic properties of cuprous oxide (Cu_2_O) have been studied extensively^19–25^. CuO_x_ has been demonstrated as an efficient p-contact for PSCs owing to the good match between its valence bands and perovskite and its promising hole mobility^26–30^. The efficiency was recently improved to 19% in a p-i-n inverted structure using a Cl-doped CH_3_NH_3_PbI_3_ perovskite^31^. However, the CuO_x_-coated substrate generally suffers a low transmittance in the visible light range below 550 nm and ultrathin CuO_x_ films are necessary to minimize the optical loss in the inverted structure. It should be noted that much less work has been implemented on inorganic HTMs for use in PSCs with n-i-p structure^10,13,32^. The deposition of HTMs on perovskite is limited because perovskite is vulnerable to high temperatures and polar solvents. In 2016, Nejand *et al*. demonstrated the production of a uniform, dense, and pinhole-free Cu_2_O-covered perovskite by controlling the tilting angle of the substrate and the deposition power and period via angular rotation of the substrate during the reactive magnetron sputtering process. This angular rotation prevents bombardment damage on the perovskite, but the efficiency of the solar cell only reached 8.93%^10^.

In this work, we report, for the first time, a p-type Cu/Cu_2_O and a spiro-OMeTAD layer as combinatorial HTMs in n-i-p heterojunction PSCs to improve the hole extraction capability, device performance, and device stability. For a 20-nm-thick Cu/Cu_2_O film with an oxygen flow ratio (OFR) of 60%, a high mobility of 60.5 cm^2^/V-s was obtained, which results in a superior PCE of 17.11% for the PSC, as compared to the standard device with only spiro-OMeTAD as the HTM (PCE of 13.97%). The stability test shows that the Cu/Cu_2_O layer provides enhanced durability under ambient conditions. Hence, the p-type Cu/Cu_2_O composite film is a promising HTM modified layer for highly efficient PSCs and a good protection layer for the perovskite active layer. In addition, to meet the demand for energy level alignments in our devices, insertion of the spiro-OMeTAD layer is necessary to prevent damage of the perovskite during the high energy sputtering of the Cu/Cu_2_O composite layer.

## Results

### Characterizations of the Cu/Cu_2_O composite films

**Figure 1:**
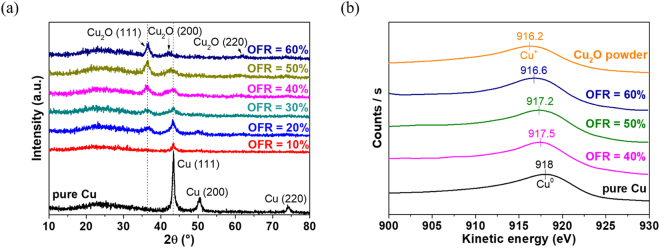
(**a**) XRD profiles of the Cu_2_O films deposited at different OFRs from 10% to 60%. The pure Cu (OFR = 0%) film is also shown for comparison. (**b**) AES of pure Cu, Cu_2_O powderand Cu/Cu_2_O composite films deposited under OFRs of 40%, 50%, and 60%.

Figure 1 shows the X-ray diffraction (XRD) patterns of the ion-beam sputtered Cu/Cu_2_O composite films deposited on glass over a 5-min period (approximately 20-nm thick) with the OFR ranging from 0 to 60%. The diffraction from pure Cu in the absence of oxygen is shown at the bottom of Fig. 1(a). The characteristic peaks at 43.4°, 50.45°, and 74.05° can be assigned to the Cu (111), (200), and (220) planes, respectively. For the OFR increasing from 10% to 50%, the intensities of the Cu phases are weaken while the Cu_2_O phase are intensified, suggesting the increasing volume ratio of Cu_2_O in the nanocomposite films. With further increasing OFR to 60%, there is no visible Cu diffraction and the Cu_2_O (220) phase dominates. The scanning electron microscopy (SEM) images of the corresponding Cu/Cu_2_O composite films are provided in the Supporting Information Fig. S1. Owing to the difference in the contrast between Cu and Cu_2_O, a small number of bright spots were observed for the OFR ranging from 30% to 50%, which are likely associated with the existence of Cu (Supporting Information Fig. S1(c–e)). Smooth morphology with a negligible number of bright spots was observed for the Cu_2_O film with an OFR of 60%, as shown in the Supporting Information Fig. S1(f), which indicates that most of Cu was oxidized to form Cu_2_O.

For convenience, the Cu/Cu_2_O composite films grown under an OFR of X% are denoted as Cu_2_O_X in the rest of the paper. To determine the elemental composition of the Cu/Cu_2_O composite films, Auger electron spectroscopy (AES) was performed to monitor the Cu LMM transition, as seen in Fig. 1(b). The peak at 918 eV corresponds to Cu^0^ bonding for an OFR of 0%. With the increase of OFR, the characteristic AES peak shifts toward lower kinetic energy (917.5 eV for Cu_2_O_40 and 917.2 eV for Cu_2_O_50), reflecting the increase of the Cu_2_O component in the composite film. For Cu_2_O_60, the characteristic peak at 916.6 eV is very close to the energy of Cu^+^ bonding in Cu_2_O powder (916.2 eV)^33^. This shows that the films produced with high OFR exhibit spectra close to that of pure Cu_2_O.

**Figure 2:**
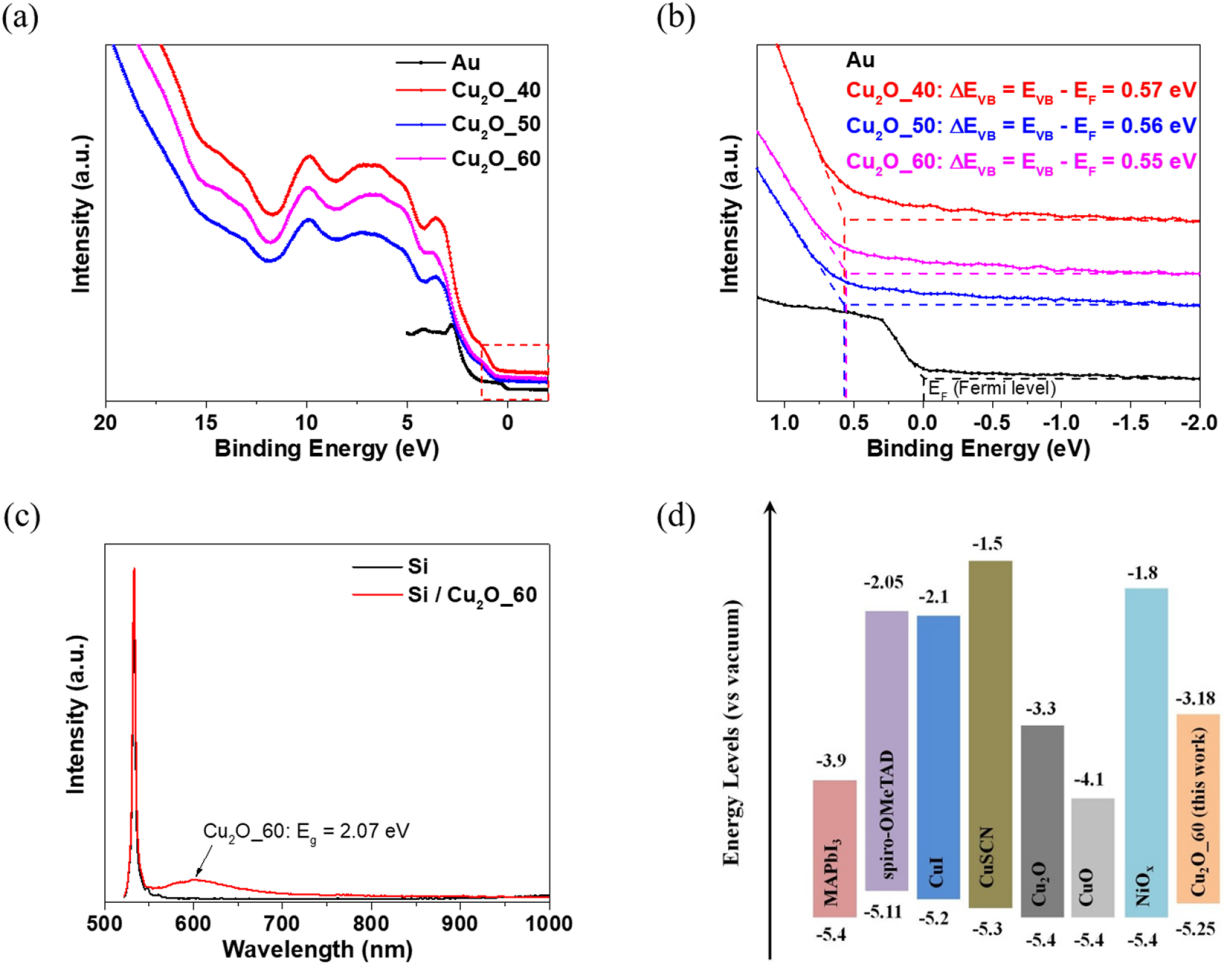
(**a**) Full UPS characterization and (**b**) UPS spectra of the valence band region near the Fermi level for Au and the Cu/Cu_2_O composite films. (**c**) PL spectrum of the Cu_2_O_60 film with a band gap of 2.07 eV. (**d**) Schematic energy level alignment of various HTMs including Cu_2_O film for comparison.

To investigate the energy level and the relative position of the Fermi level with respect to the valence band maximum (∆E_VB_) of the Cu/Cu_2_O composite films, ultraviolet photoelectron spectroscopy (UPS) and photoluminescence (PL) spectroscopy were performed, as shown in Fig. 2. Figure 2(a) shows the full UPS spectra and Fig. 2(b) shows a magnification of the spectra near the valence band region from Fig. 2(a). The Au metal rectifies the Fermi energy level position at 0 eV, and the obtained ∆E_VB_ of the Cu_2_O_60 film is 0.55 eV. The corresponding band gap (E_g_) determined from the PL spectra (Fig. 2(c)) is 2.07 eV and the work function of Au is approximately 4.7 eV^34^. The peak values in Fig. 2(b) correspond to the energies between the valence band maximum (VBM) and the work function, and the VBM position of the Cu_2_O_60 was determined to be approximately −5.25 eV. The conduction band minimum (CBM) of the Cu_2_O_60 is −3.18 eV that was determined by the addition of E_g_ to the VBM. The energy level of the Cu_2_O_60 film along with other commonly-used inorganic HTMs is plotted in Fig. 2(d) for comparison. It is noted that the VBM of Cu/Cu_2_O film is compatible with the other HTMs, suggesting that Cu/Cu_2_O film can effectively extract the hole carrier from perovskite.

The electrical properties of the Cu/Cu_2_O composite films were analyzed by Hall effect measurements and a four-point probe method, and are summarized in Table 1. Figure S2 presents the Hall effect measurements of Cu/Cu_2_O composite films at variant OFRs. The Cu_2_O_40, Cu_2_O_50, and Cu_2_O_60 films have been determined to be p-type semiconducting materials. As evidenced from the Hall effect measurements shown in Fig. S2(a), for OFR = 30% the Cu/Cu_2_O composite film is a n-type semiconductor, which is not of interest for HTM application. The Cu_2_O_60 film is nearly insulating (sheet resistance ~83 kΩ/square) owing to the high volume ratio of Cu_2_O. The sheet resistances of the Cu_2_O_40 and Cu_2_O_50 films are 72.5 and 210.1 Ω/square, respectively, and the corresponding hole mobilities are 1.87 and 1.23 cm^2^/V-s, which are at least three orders of magnitude higher than the organic hole transport layers of spiro-OMeTAD (1.6 × 10^−4^~1.6 × 10^−3^ cm^2^/V-s). Notably, the hole mobility of the Cu_2_O_60 film is as high as 60.5 cm^2^/V-s. The hole carrier concentrations of the Cu_2_O_40, Cu_2_O_50, and Cu_2_O_60 films are 2.3 × 10^22^, and 6.19 × 10^17^ cm^−3^, respectively.

**Table 1:** Electrical properties of the Cu/Cu_2_O films under different oxygen flow ratios (OFRs) and that of spiro-OMeTAD for comparison.

OFR (%)	Thickness (nm)	Type	Sheet Resistance (Ω/square)	Mobility (cm^2^/V-s)	Bulk Concentration (cm^−3^)
40	20	p	72.5	1.87	2.3 × 10^22^
50	20	p	210.1	1.23	1.54 × 10^22^
60	20	p	83360	60.5	6.19 × 10^17^
spiro-OMeTAD/tBP/LiTFSI	100–200	p		1.6 × 10^−4^ 1.6 × 10^−3^	

**Figure 3:**
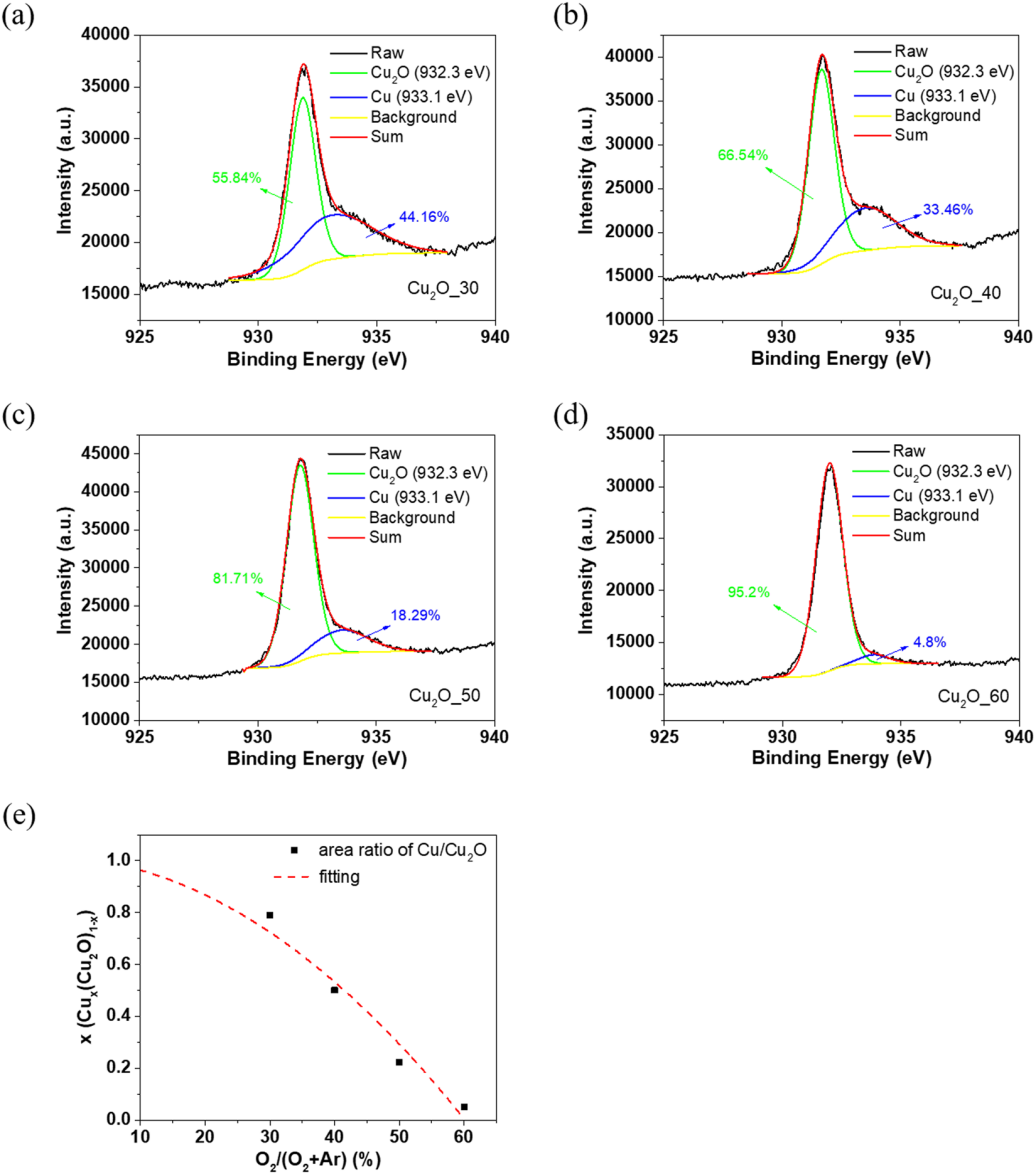
Cu 2p_3/2_ XPS spectra of (**a**) Cu_2_O_30, (**b**) Cu_2_O_40, (**c**) Cu_2_O_50, and (**d**) Cu_2_O_60 films. (**e**) Plot of Cu content vs. oxygen flow ratios obtained by deconvoluting XPS peak and integrating corresponding area. The dashed curve is a guide for eyes.

X-ray photoelectron spectroscopy (XPS) was employed to analyze the chemical compositions of the composite Cu/Cu_2_O films grown with different OFRs. Figure 3(a–d) show the XPS spectra of Cu 2p_3/2_ core level. All the films exhibit characteristic peaks at 932.3 eV and 933.1 eV that are respectively corresponding to copper(I) oxide and copper metal as reported in the literature^35,36^. By deconvoluting the signal and integrating the individual area, the ratio of Cu to Cu_2_O for Cu_2_O_30, Cu_2_O_40, Cu_2_O_50 and Cu_2_O_60 films are estimated to be 0.79, 0.50, 0.22 and 0.05, respectively. The Cu/Cu_2_O ratio as a function of oxygen flow ratios is shown in Fig. 3(e), indicating that the Cu content decreases with the oxygen flow. The tendency of Cu content in the sputtered film is consistent with the SEM results.

### Devices

**Figure 4:**
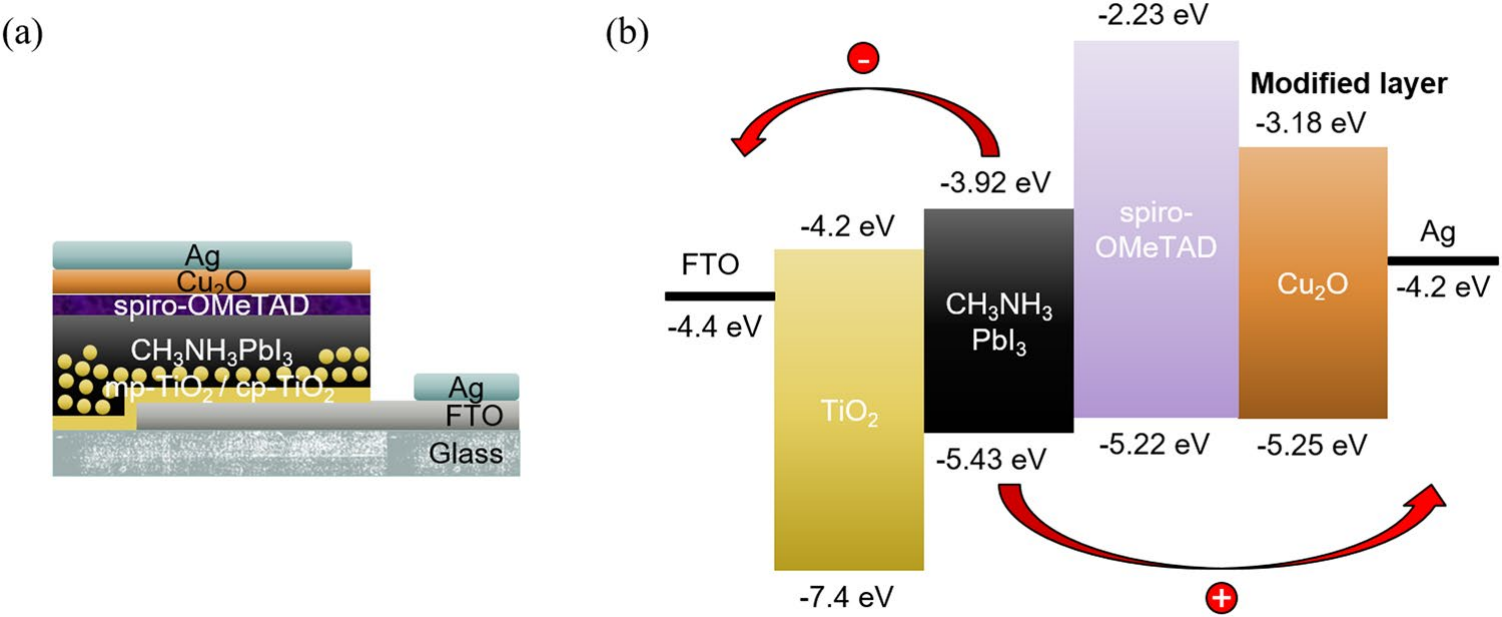
(**a**) Device illustration and (**b**) the corresponding band diagram of the general device architecture.

The device architecture is based on the n-i-p heterojunction configuration which is composed of FTO/cp-TiO_2_/mp-TiO_2_/CH_3_NH_3_PbI_3_/spiro-OMeTAD/Cu_2_O_X/Ag, as shown in Fig. 4(a). The standard (reference) device is the one without the Cu_2_O_X film interlayer. The active layer of the CH_3_NH_3_PbI_3_ perovskite was prepared by solvent engineering^37^, while the Cu_2_O_X interlayer was grown by sputtering on a Cu target by tuning the OFR over a 5 min period. The thickness of the Cu_2_O_X films are approximately 20 nm, as estimated by the cross-sectional SEM images (cf. Fig. S1 in Supporting Information). For convenience and clarity, the energy level diagram of the device is depicted in Fig. 4(b).

**Figure 5:**
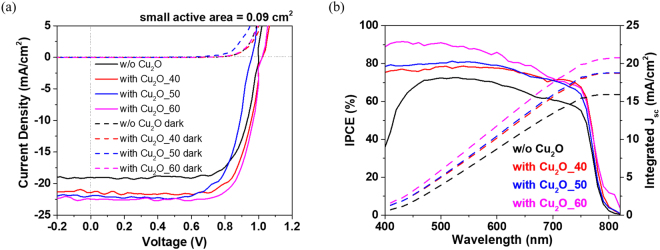
(**a**) J-V characteristics of perovskite solar cells without and with Cu_2_O_X (X = 40, 50 and 60) for active area of 0.09 cm^2^. (**b**) IPCE curves and integrated current density for corresponding PSCs.

Figure 5(a) shows the current density-voltage (J-V) curves of the Cu_2_O_X based perovskite solar cells (with active area of 0.09 cm^2^) prepared with varying OFRs, and the corresponding photovoltaic parameters are summarized in Table 2. Amongst these devices, the Cu_2_O_60 device had a short circuit current density (J_sc_) of 22.46 mA/cm^2^, open circuit voltage (V_oc_) of 1.03 V, and fill factor (FF) of 74.1%, resulting in the best PCE of 17.11% under AM 1.5 G illumination. Produced under similar fabrication conditions, the PSCs incorporating Cu_2_O_40 and Cu_2_O_50 layers show J_sc_ of 21.52 and 21.97 mA/cm^2^, V_oc_ of 1.03 and 0.95 V, FFs of 73.18 and 72%, and PCEs of 16.21 and 14.98%, respectively. The reference device without Cu_2_O_X showed a J_sc_ of 19.02 mA/cm^2^, V_oc_ of 0.997 V, FF of 73.63%, and PCE of 13.97%. The incorporation of the Cu_2_O_X interlayer serves as a bridge between the organic HTM (spiro-OMeTAD) and the metal electrode (Ag), and facilitates hole transport in the device owing to the high hole conductivity of the Cu/Cu_2_O composite films. Notably, the electron mobility of TiO_2_ ranges from 0.1–10 cm^2^/V-s^38,39^, while the hole mobility of Cu_2_O_X is typically between 1–60.5 cm^2^/V-s. Unbalanced ambipolar transport may be the main cause of the hysteresis observed in the prepared devices^40–42^. The introduction of spiro-OMeTAD not only protects the perovskite active layer during sputtering deposition but also acts as an electron blocking layer, as schematically illustrated in Fig. 4(b). Figure 5(b) shows the incident photon-to-electron conversion efficiency (IPCE) spectra of the four fabricated devices. The devices with Cu_2_O_40 and Cu_2_O_50 layers show good responses with maximum values of approximately 80% in the range of 400 to 700 nm. The IPCE is approximately 90% for the Cu_2_O_60 incorporated device over the same wavelength range. The IPCE spectra reveal that the Cu_2_O_40, Cu_2_O_50, and Cu_2_O_60 incorporated devices yield higher responses and photocurrents than the standard device. Figure S3 (Supporting Information) shows the statistical distribution of the photovoltaic parameters resulted from 15 devices for Cu_2_O_60-based PSCs as well as 15 standard devices for comparison. The results show that the Cu_2_O_60-based device exhibits better efficiency than the reference devices. We further enlarge active area of 2.06 cm^2^ for Cu_2_O_60 based perovskite solar cell which exhibits a J_sc_ of 21.66 mA/cm^2^, V_oc_ of 1.0 V, FF of 42.47%, and PCE of 9.22% and its corresponding J-V curve is shown in Supporting Information Fig. S4. It can be found that the device with large 2.06 cm^2^ active area is lower than that of the smaller 0.09 cm^2^ active area ones due to the poor FF. The reducing FF is mainly resulted from the increasing sheet resistance of FTO substrate that raises the probability of the charge recombination before carrier extracted by electrode^43,44^.

**Table 2:** Photovoltaic parameters of the Cu_2_O-based perovskite solar cells with varying OFRs. The standard spiro-OMeTAD device without Cu_2_O is shown for comparison.

Device (0.09 cm^2^)	V_oc_ (V)	J_sc_ (mA/cm^2^)	FF (%)	PCE (%)	Rs (Ω)	Rsh (Ω)
w/o Cu_2_O	0.997	19.02	73.63	13.97	48.81	14.8 k
Cu_2_O_40	1.03	21.52	73.18	16.21	79.13	16.36 k
Cu_2_O_50	0.95	21.97	72.0	14.98	53.99	2.77 k
Cu_2_O_60	1.03	22.46	74.1	17.11	60.68	6.95 k

### Stability Test

**Figure 6:**
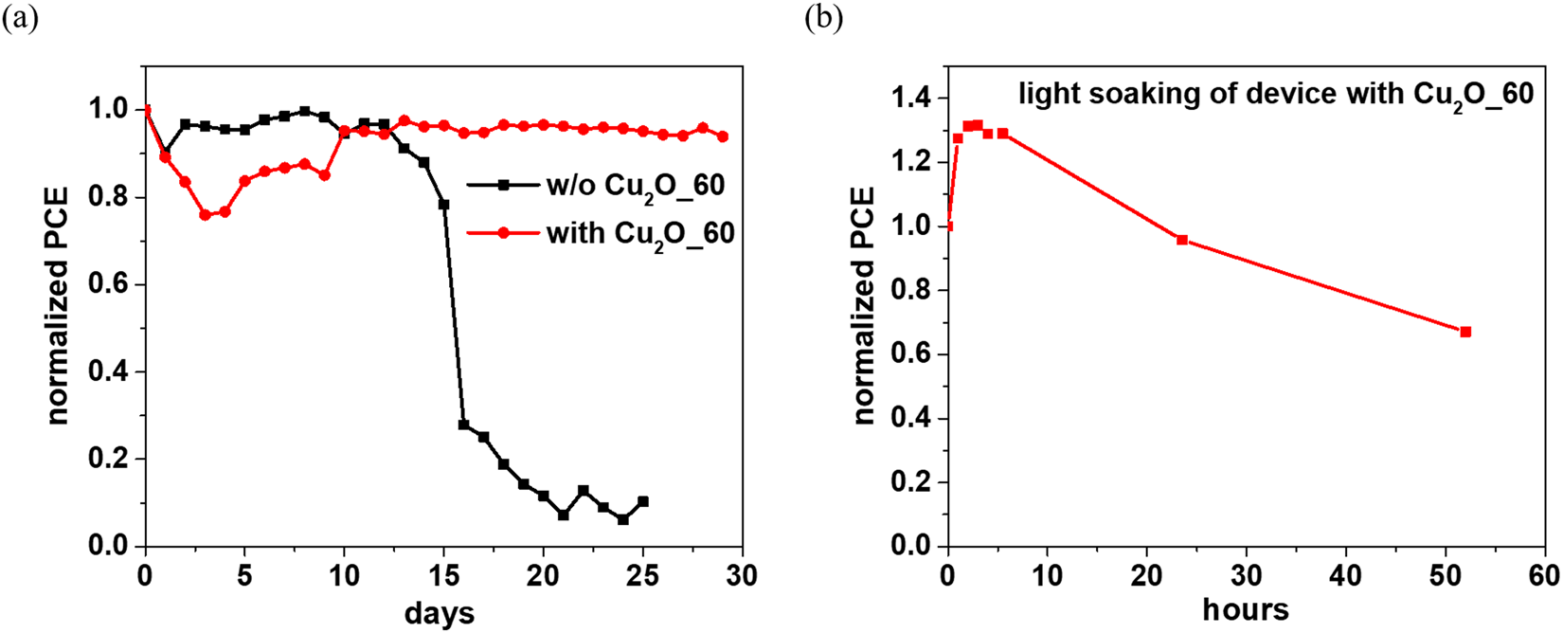
(**a**) Stability test of the encapsulated devices without Cu_2_O_60 and with Cu_2_O_60 under dark conditions. (**b**) Stability test of the device with Cu_2_O_60 under continuous lighting.

Finally, we monitor the long-term device stability of the normalized efficiency for the encapsulated Cu_2_O_60-based devices along with the standard cell and the results are shown in Fig. 6(a). When stored in the dark, the efficiency of the standard device drops rapidly after 12 days, while the devices with the Cu/Cu_2_O composite films show long-term stability for at least 30 days. The results demonstrate that p-type Cu/Cu_2_O composite films are promising candidates for the modification of the HTM property for high efficiency PSCs, while simultaneously acting as a protecting layer. The devices with the Cu/Cu_2_O composite film were transferred to a light soaking condition (one sun illumination). The aging test indicated that approximately 67% of the initial efficiency is retained after 50 h, as shown in Fig. 6(b).

## Discussion

In summary, p-type Cu/Cu_2_O composite films with a tunable Cu/Cu_2_O ratio were fabricated by controlling the oxygen flow rate during the ion beam sputtering of Cu targets. The resistance and mobility of the Cu/Cu_2_O composite films can be controlled to optimize the device performance of the PSCs with an n-i-p heterojunction configuration. In comparison to the standard device, the PCEs of the devices incorporating Cu/Cu_2_O composite films are significantly improved. Furthermore, the Cu/Cu_2_O composite films can effectively protect the perovskite active layer and enhance the lifetime of the PSCs.

## Methods

### Fabrication of standard perovskite solar cells

The fluorine-doped tin oxide (FTO) glass was etched with zinc powder and hydrochloric acid (1 M) to house two electrodes, which were cleaned by an ultrasonic soap, water, and ethanol bath and then rinsed with deionized water. A 70-nm-thick TiO_2_ compact layer (cp-TiO_2_) was deposited on the cleaned FTO substrates by spray pyrolysis of a titanium diisopropoxide bis(acetylacetonate) (75 wt.% in isopropanol, Aldrich) precursor solution diluted in ethanol (1:39 v:v), at 485 °C for 30 min. This was followed by the deposition of a 20 mM TiCl_4_ solution layer, which functions as an electron transport layer (ETL). The mesoporous TiO_2_ layer (mp-TiO_2_) was formed by spin-coating a diluted TiO_2_ solution (1:8.5 v:v) at 4000 rpm for 30 s followed by sintering at 500 °C for 30 min. The CH_3_NH_3_PbI_3_ precursor was prepared inside a nitrogen-filled glove box with oxygen and moisture levels <1 ppm by mixing 0.288 g of CH_3_NH_3_I (dyesol, 99.99%) with 0.831 g of PbI_2_ (Aldrich, 99.999%, ultra-dry) in a 1:1 equimolar ratio. The components were dissolved in a mixture of γ-butyrolactone (GBL) and dimethyl sulfoxide (DMSO) (6:4 v:v) and heated at 50 °C under stirring for 12 h for complete dissolution. CH_3_NH_3_PbI_3_ was then spin-coated onto the FTO/cp-TiO_2_/mp-TiO_2_ substrate at 1000 rpm for 10 s and at 5000 rpm for 30 s, and the anti-solvent toluene was injected for 15 s in the second spin step. The spin-coated perovskite film was dried at 100 °C for 10 min to remove the toluene. The spiro-OMeTAD solution was prepared by dissolving 72.3 mg of spiro-OMeTAD, 28.8 μL of tert-butylpyridine (tBP), and 17.5 μL of lithium-bis(trifluoromethanesulfonyl)imide (Li-TFSI) (from a stock solution of 520 mg/mL of Li-TFSI in acetonitrile (Aldrich, 99.8%)) in 1 mL of chlorobenzene. The spiro-OMeTAD solution was then spin coated onto the MAPbI_3_ substrate at 4000 rpm for 20 s. Finally, a 60-nm-thick silver layer was thermally evaporated to form the counter electrode.

### Fabrication of Cu_2_O-based perovskite solar cells

The MAPbI_3_ substrates were fabricated as described above. The spiro-OMeTAD serves as a buffer layer, and an ion beam sputtering system was used to deposit the Cu/Cu_2_O films. The vacuum chamber was evacuated to a base pressure of 2.2 × 10^−6^ torr. A copper substrate (99.99% purity) was used as the sputtering target. The Cu/Cu_2_O films were grown at room temperature (~30 °C) with different Ar and O_2_ flow rates.

### Characterization

The crystallographic properties of the films were determined by grazing incidence X-ray diffraction using Cu Kα radiation (λ = 1.5418 Å, D8, Bruker, Germany) at room temperature with a scanning step size of 0.005°. The surface morphology and cross-section of the samples were analyzed by field-emission scanning electron microscopy (SUPRA^TM^ 55). The carrier type, carrier concentration, and electrical resistivity were measured using a four-terminal van der Pauw configuration at room temperature. The UPS and XPS experiment were performed at beamline 24 A of the Taiwan Light Source in the National Synchrotron Radiation Research Center (NSRRC). A microscopic PL system (MRI, Protrustech Co., Ltd., Taiwan) was used to determine the band gap of the films with a pumping wavelength of 532 nm. The J-V measurement was performed using a solar simulator (SS-F5-3A, Enlitech) with AM 1.5 G spectra and the device was connected to a source meter (Keithley 2401) for recording the J-V data. The light intensity was calibrated using reference silicon solar cells to be 100 mW/cm^2^. The scan rate was 1 V/s for the forward scan (from J_sc_ to V_oc_). A metal mask with an aperture size of 0.09 cm^2^ was used to define the active area. A 300 W intensity monochromatic (Newport Cornerstone 260) xenon lamp (Newport) and a source meter (Keithley 2401) were integrated to measure the IPCE response of the devices.

## Electronic supplementary material

Supplementary Information
