# Supplementary material for: Cu/Cu2O nanocomposite films as a p-type modified layer for efficient perovskite solar cells
Source: Sci Rep. 2018 May 16;8:7646. doi: 10.1038/s41598-018-25975-8 (PMC5955939; doi:10.1038/s41598-018-25975-8)
Supplement: Supplementary file 1 — Supplementary Information [file 41598_2018_25975_MOESM1_ESM.docx]

**Supporting Information**

Cu/Cu_2_O nanocomposite films as a p-type modified layer for efficient perovskite solar cells

You-Jyun Chen^1^, Ming-Hsien Li^1^, Jung-Chun-Andrew Huang^2,4^*, and Peter Chen^1,3,5^*

^1^Department of Photonics, National Cheng Kung University, Tainan 701, Taiwan

^2^Department of Physics, National Cheng Kung University, Tainan 701, Taiwan

^3^Center for Micro/Nano Science and Technology (CMNST), National Cheng Kung University, Tainan, 701, Taiwan

^4^Quantum Topology Center (QTC), National Cheng Kung University, Tainan, 701, Taiwan

^5^Hierarchical Green-Energy Materials Research Center, National Cheng Kung University, Tainan, 701, Taiwan

**Corresponding Authors**

E-mail addresses: [jcahuang@mail.ncku.edu.tw](mailto:jcahuang@mail.ncku.edu.tw) (J.-C. Huang), [petercyc@mail.ncku.edu.tw](mailto:petercyc@mail.ncku.edu.tw) (P. Chen).


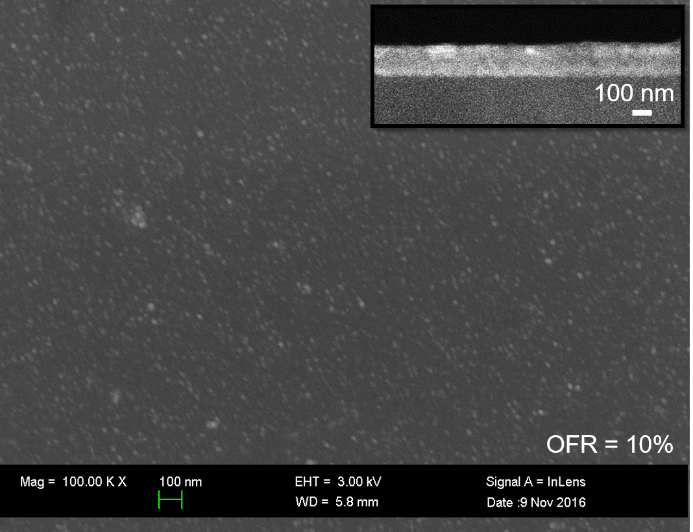

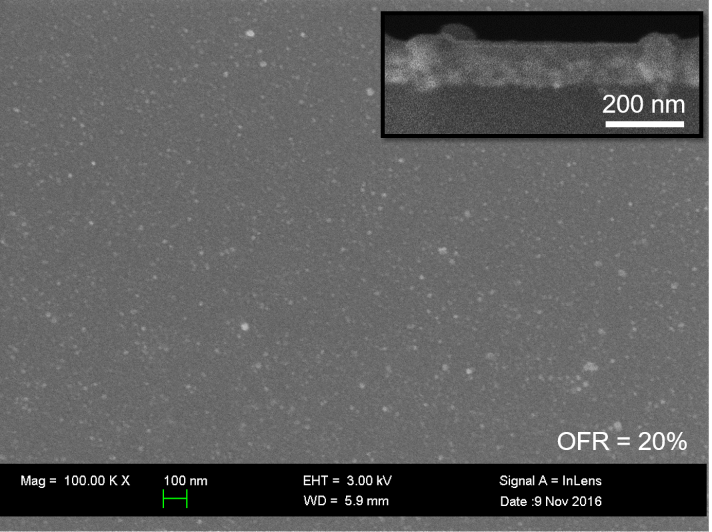


(b)

(a)


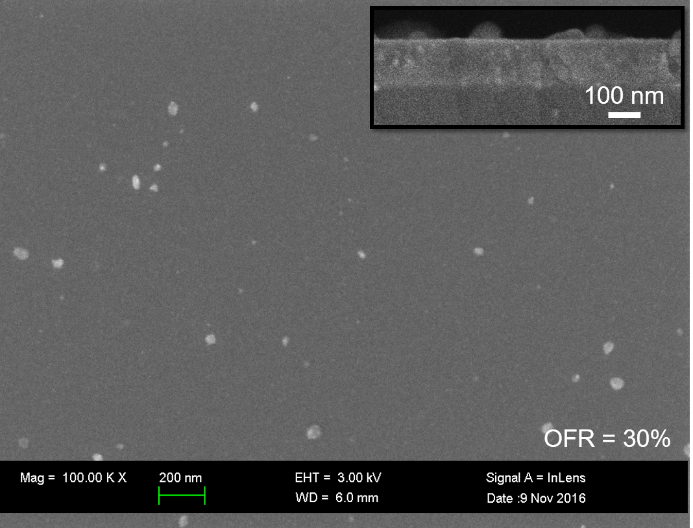

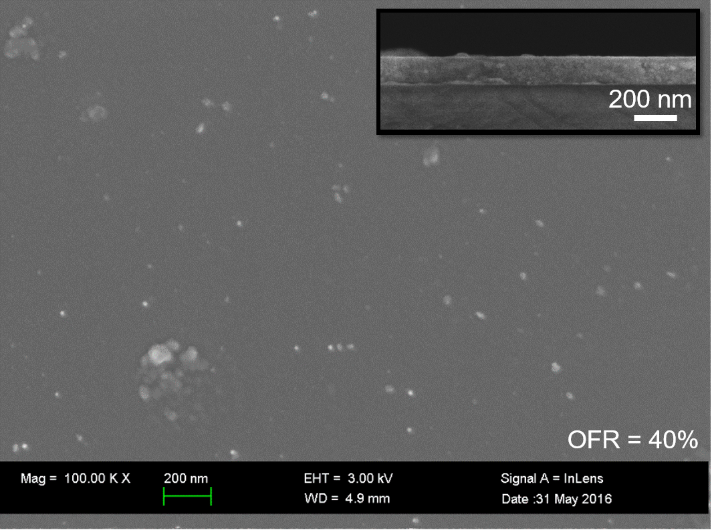


(d)

(c)


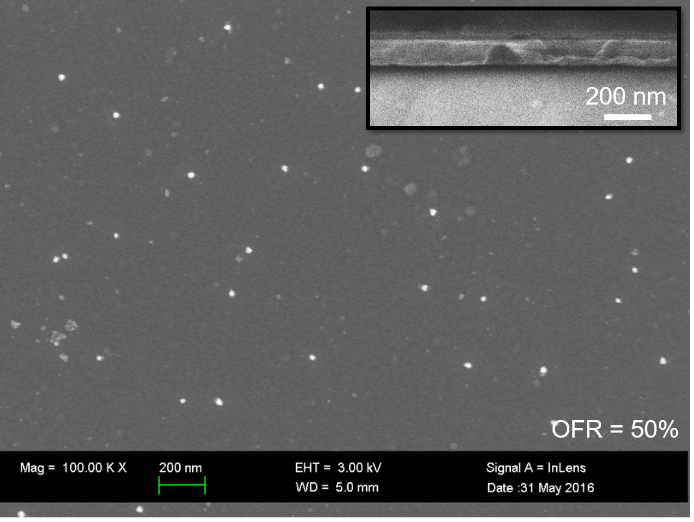

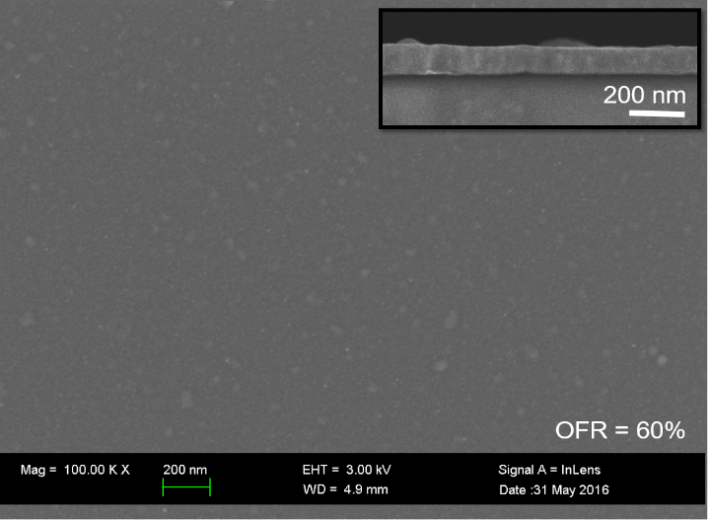


(f)

(e)

**Figure S1.** SEM images of the top-view of Cu/Cu_2_O composite films with different oxygen flow ratios (a) 10 %, (b) 20 %, (c) 30 %, (d) 40 %, (e) 50 %, and (f) 60 %. Insets show the cross-sectional SEM images.


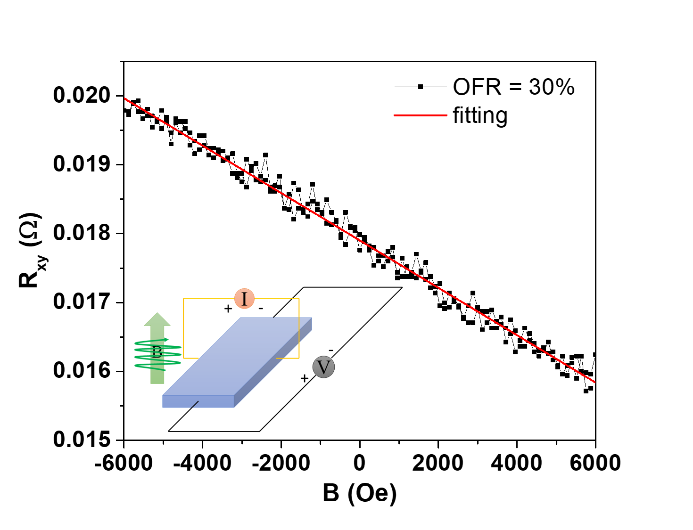

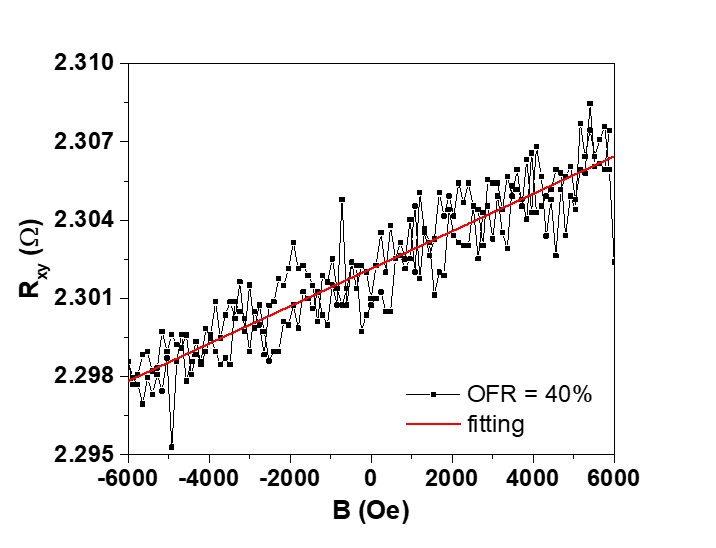


(a)

(b)


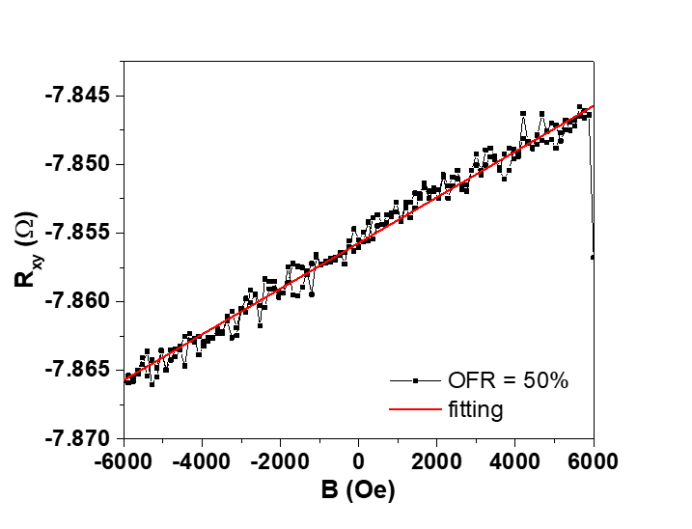

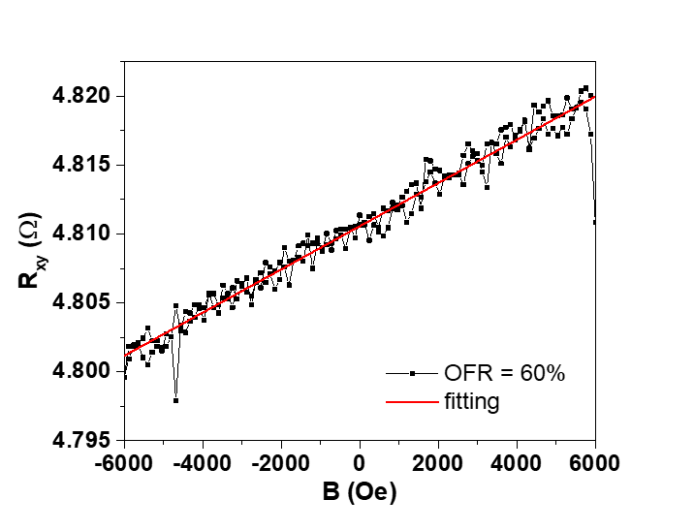


(c)

(d)

**Figure S2.** Hall effect measurements of the Cu/Cu_2_O films with oxygen flow ratio of (a) 30%, (b) 40%, (c) 50%, and (d) 60%. Inset of (a) shows the input orientation of voltage, current, and magnetic field.


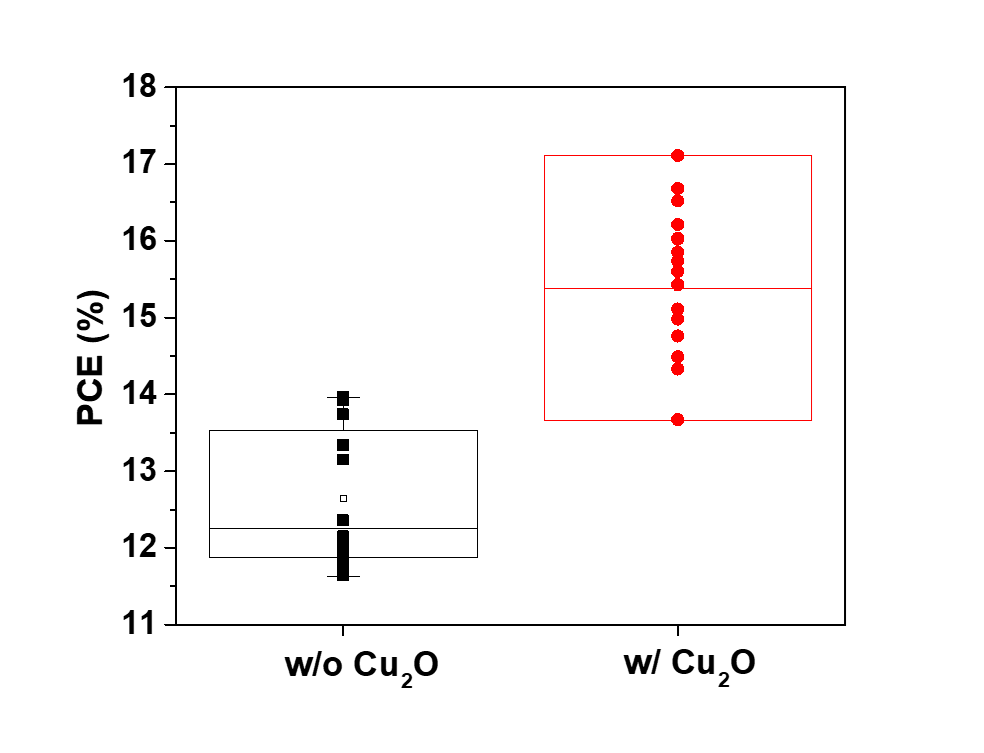

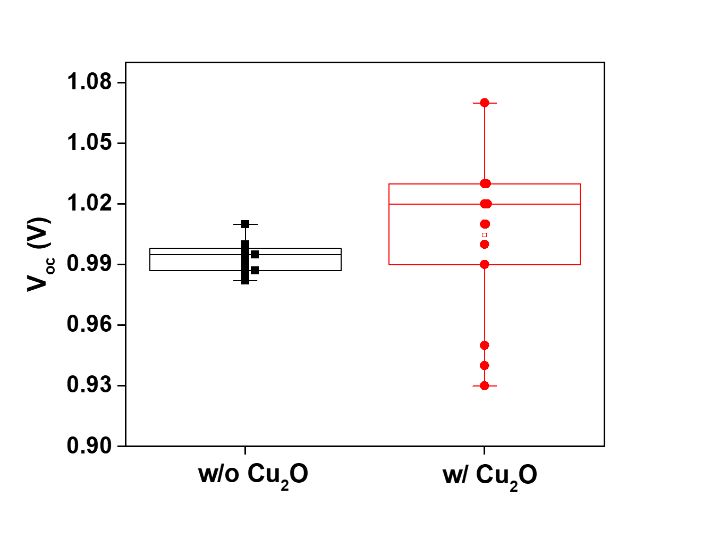


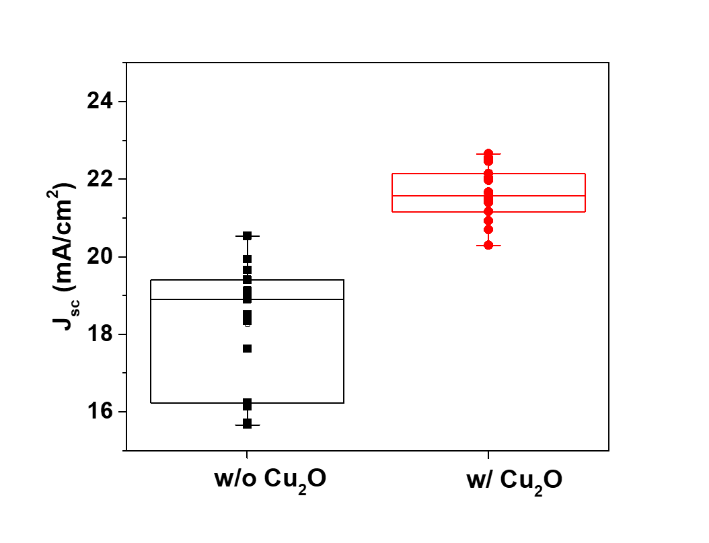

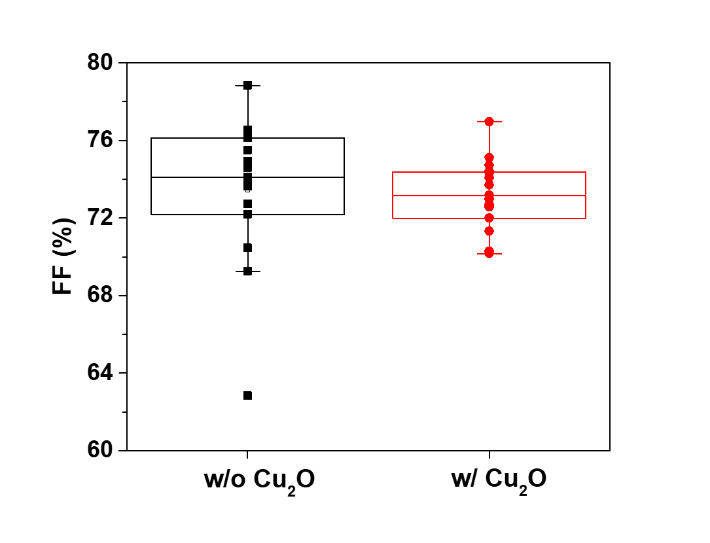


**Figure S3.** Statistical efficiency diagram of 15 cells without Cu_2_O and with Cu_2_O-based devices.


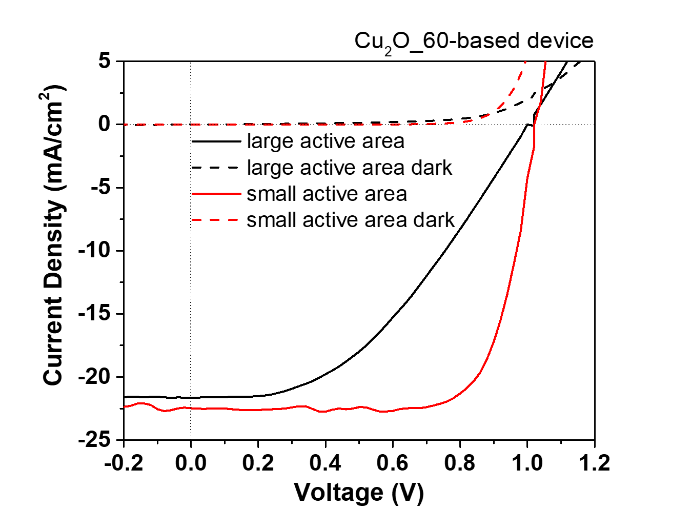


**Figure S4.** JV characteristics of Cu_2_O_60 based perovskite solar cells with a large active area 2.06 cm^2^ and a small active area 0.09 cm^2^.
